# Neighborhood Opportunity, Hospital Volume, and Pediatric Postoperative Mortality

**DOI:** 10.1001/jamanetworkopen.2025.43017

**Published:** 2025-11-12

**Authors:** Sandra Tay, Christian Mpody, Joseph D. Tobias, Brittany L. Willer

**Affiliations:** 1 Department of Anesthesiology and Pain Medicine, Nationwide Children’s Hospital, Columbus, Ohio; 2 Department of Anesthesiology, Montefiore Medical Center, Bronx, New York; 3 Department of Anesthesiology, The Ohio State University, Columbus

## Abstract

**Importance:**

Sociodemographic disparities in pediatric perioperative care are often associated with residential segregation, referral patterns, and the regionalization of quaternary centers, which can concentrate patients from disadvantaged neighborhoods in low-volume hospitals. Although neighborhood conditions are increasingly recognized as critical to child health, their interaction with hospital volume and postoperative mortality remains unclear.

**Objectives:**

To assess the association of neighborhood opportunity with pediatric postoperative mortality, and whether this association is modified by hospital volume.

**Design, Setting, and Participants:**

This retrospective cohort study used the multicenter administrative Pediatric Health Information System (PHIS) database from January 1, 2012, to September 30, 2024. The study comprised patient encounters among children younger than 18 years who underwent inpatient surgical procedures at a PHIS-reporting hospital.

**Exposure:**

Neighborhood opportunity, as measured by the Child Opportunity Index 3.0, modified by hospital volume.

**Main Outcomes and Measures:**

Postoperative in-hospital mortality.

**Results:**

Of 996 865 inpatient surgical encounters among 821 865 children (mean [SD] age, 7.2 [5.9] years), 55.7% of encounters were among boys, 16.0% were among children who resided in neighborhoods of very high opportunity, 17.9% in neighborhoods of high opportunity, 18.0% in neighborhoods of moderate opportunity, 21.1% in neighborhoods of low opportunity, and 27.0% in neighborhoods of very low opportunity. In addition, 71.8% of patients were treated at high-volume centers, and 28.2% at low-volume centers. The in-hospital postoperative mortality rate was 0.9%, with higher rates observed among children from lower opportunity areas. Compared with children from very high opportunity neighborhoods, increased mortality risk was associated with decreasing neighborhood opportunity, reaching a 27% higher risk in very low opportunity areas (adjusted risk ratio [ARR], 1.27; 95% CI, 1.18-1.38; *P* < .001). Mortality rates also varied by hospital volume, with rates of 0.88% at high-volume centers and 0.92% at low-volume centers. Care at low-volume centers was associated with an 11% higher risk of in-hospital mortality compared with high-volume centers (ARR, 1.11; 95% CI, 1.06-1.16*; P* < .001); however, hospital volume did not modify the association between neighborhood opportunity and mortality.

**Conclusions and Relevance:**

In this cohort study, higher neighborhood opportunity was associated with lower risk of in-hospital postoperative mortality among pediatric patients. Although care at high-volume hospitals is typically associated with improved outcomes, this association was not observed for children from disadvantaged neighborhoods. These findings suggest that, beyond hospital-level interventions, addressing area-level social drivers of health may be essential to improving surgical outcomes in pediatric populations.

## Key Points

**Question:**

Are neighborhood opportunity and hospital volume associated with risk of postoperative mortality among pediatric patients?

**Findings:**

In this cohort study of more than 996 000 pediatric surgical encounters, children from lower-opportunity neighborhoods faced a greater risk of postoperative mortality than those from higher-opportunity areas. Although children from higher-opportunity neighborhoods treated at high-volume hospitals had the lowest mortality risk, this volume-outcome association was not observed among children from lower-opportunity neighborhoods.

**Meaning:**

This study suggests that children from disadvantaged neighborhoods had a greater risk of postoperative mortality; treatment at high-volume hospitals was not associated with lower risk for these children.

## Teaser

This cohort study uses the Pediatric Health Information System (PHIS) database to assess the association of neighborhood opportunity and hospital volume with risk of postoperative mortality among pediatric patients.

## Introduction

Sociodemographic disparities in health care contribute to the persistent reality that individuals from marginalized communities are more likely to die after surgery. In the pediatric setting, this mortality gap is particularly stark. Children of minority race and ethnicity, those from low-income households, and those whose language of care is other than English face disproportionately higher risks of postoperative death.^1,2,3^ These outcomes are not solely the result of individual clinical factors but reflect broader systemic failures. Residential segregation, inequities in specialty referral, and the regionalization of high-volume surgical centers mean that socially disadvantaged children are often funneled into lower-volume hospitals—facilities that may lack the infrastructure or health care professionals with the experience to deliver optimal surgical care.^4,5,6,7^ As a result, structural inequities become life-threatening, manifesting as preventable mortality among the most vulnerable pediatric patients.

Neighborhoods are increasingly recognized as central to child health equity, shaping access to resources essential for healthy development. The Child Opportunity Index captures this “neighborhood opportunity” through measures of environmental exposure, quality of housing, education, and economic resources, as well as access to transportation and health care.^8^ Lower neighborhood opportunity has been associated with several pediatric health outcomes, such as cardiometabolic risk, asthma, acute care utilization, and emergency and hospital readmissions.^9^ However, to our knowledge, the association of neighborhood opportunity with pediatric postoperative mortality remains to be studied.

The objective of this study was to assess the association of neighborhood opportunity with pediatric postoperative mortality and whether this association is modified by hospital volume. We hypothesized that decreasing neighborhood opportunity would be associated with increased postoperative mortality risk. We further hypothesized that undergoing surgery at a high-volume center would attenuate this association.

## Methods

### Study Design and Data Source

We conducted a retrospective cohort study using the Pediatric Health Information System (PHIS) database, a robust administrative database containing deidentified inpatient, emergency, ambulatory, and observation unit encounters from approximately 50 US pediatric hospitals affiliated with the Children’s Hospital Association. The database includes patient demographics, admission and discharge information, and diagnoses and procedures, coded using *International Classification of Diseases, Ninth Revision, Clinical Modification*, *International Statistical Classification of Diseases and Related Health Problems, Tenth Revision, Clinical Modification*, and *Current Procedural Terminology* codes. All data are deidentified and undergo extensive reliability and validity checks to ensure data quality.^10^ This study was approved by the Nationwide Children’s Hospital institutional review board with a waiver of informed consent because the study used deidentified data. This study followed the Strengthening the Reporting of Observational Studies in Epidemiology (STROBE) reporting guideline.

### Study Population

We included children younger than 18 years of age who underwent inpatient surgery between January 1, 2012, and September 30, 2024. We excluded encounters from hospitals with data quality issues related to zip code or race and ethnicity for each quarter of data collection. In addition, patients who were transferred to another health care institution were excluded, as their primary outcomes could not be fully observed during their hospital stay.

### Variables

The primary outcome was in-hospital postoperative mortality, defined as in-hospital death occurring during the same encounter as an index surgery. The main exposure was neighborhood opportunity; hospital volume was considered a potential effect modifier. Neighborhood opportunity was measured using the Child Opportunity Index 3.0, a validated zip code–level metric that classifies neighborhoods into 5 quintiles of opportunity—very low, low, moderate, high, and very high—according to the social drivers of health inherent to the neighborhood.^8^ Hospital volume was defined as the mean annual number of procedures performed at each hospital within a given procedural group over the entire study period. For each hospital, total procedure counts were summed across all study years and divided by the number of years.^6,11^ Hospitals were classified as high- or low-volume based on whether their mean volume was above or below the median across all hospitals. We adjusted analyses for potential confounders defined a priori based on literature and clinical relevance.^1,12,13,14^ These potential confounders included age, sex, race and ethnicity, admission type, insurance type, preoperative complex chronic conditions, procedural group, and year of procedure. Age was categorized into 4 groups: infant (<1 year), young child (1-4 years), child (5-12 years), and adolescent (13-17 years). Race and ethnicity data in the PHIS database were collected via self-reports in electronic health records. We included Hispanic, non-Hispanic Asian (hereafter, *Asian*), non-Hispanic Black (hereafter, *Black*), and non-Hispanic White (hereafter, *White*) children. Patients identifying as American Indian or Alaska Native, Pacific Islander, or multiracial were excluded due to small sample sizes and heterogeneity. We treated race and ethnicity as social stratification variables without biological basis, recognizing their potential to influence a child’s health through structural racism. Preoperative chronic conditions were coded as binary variables using established classification systems.^15^ We included admission type in the analysis as a limited indicator for illness severity. Admission type was recorded in the PHIS database as elective, urgent, emergency, and trauma. Procedural groups were based on PHIS service line classifications, which use All-Patient Refined Diagnosis-Related Groups to categorize procedures into clinical areas of surgical care: cardiovascular, digestive or metabolic, hematology or oncology, neonatology, neurology, orthopedic or joint disease, respiratory, transplant, and other surgical groups.

### Statistical Analysis

Demographic and clinical characteristics were summarized based on neighborhood opportunity levels. Continuous variables were reported as median (IQR) values, and categorical variables as frequencies with percentages. We assessed multicollinearity among covariates using variance inflation factors; all variance inflation factor values ranged from 1.0 to 1.2, well below the conventional threshold of 10, indicating no concerning collinearity.^16^ To examine the association between postoperative mortality, neighborhood opportunity, and hospital volume, we used population-averaged generalized estimating equation models with an exchangeable correlation structure, clustering on patient ID to account for repeated surgical encounters within the same child.^17^ A Poisson distribution with a log-link function and robust standard errors was used to estimate adjusted risk ratios (ARRs). Interaction between neighborhood opportunity and hospital volume was assessed using likelihood ratio tests. To further explore potential differences, we conducted stratified analyses by neighborhood opportunity levels, categorized into advantaged (high and very high opportunity) and disadvantaged (low, very low, and moderate opportunity), consistent with approaches used in other literature.^18,19^ Additive interaction was evaluated using the relative excess risk due to interaction (RERI). To assess the robustness of our findings, we performed sensitivity analyses using a generalized estimating equation model clustered by hospital ID to account for potential within-hospital correlation. We calculated E-values to evaluate the potential influence of unmeasured confounding. The E-value represents the minimum strength an unmeasured confounder would need with both exposure and outcome, after adjustment, to explain the observed association. An E-value of 1 indicates no robustness; higher values suggest lower likelihood that unmeasured confounding fully accounts for the association.^20^ As an exploratory analysis, we examined temporal trends in postoperative mortality by neighborhood opportunity and hospital volume by plotting annual mortality rates over the study period. All statistical analyses were performed using R, version 4.3.0 (R Project for Statistical Computing). All *P* values were from 2-sided tests and results were deemed statistically significant at *P* < .05.

## Results

### Characteristics of Study Participants

We included 996 865 inpatient surgical encounters (55.7% [n = 555 281] among boys and 44.3% [n = 441 584] among girls; 3.4% [n = 33 691] among Asian children, 14.3% [n = 142 994] among Black children, 26.4% [n = 263 455] among Hispanic children, and 55.8% [n = 556 725] among White children) among 821 865 children (mean [SD] age, 7.2 [5.9] years) treated at PHIS-participating hospitals between 2012 and 2024 (Table). Of these encounters, 16.0% (n = 159 457) were among children who resided in neighborhoods of very high opportunity, 17.9% (n = 178 698) were among children who resided in neighborhoods of high opportunity, 18.0% (n = 179 024) were among children who resided in neighborhoods of moderate opportunity, 21.1% (n = 210 236) were among children who resided in neighborhoods of low opportunity, and 27.0% (n = 269 450) were among children who resided in neighborhoods of very low opportunity. Most Asian children (n = 33 691) lived in very high opportunity areas (39.0% [13 147]), with only 11.9% (n = 4000) in very low opportunity neighborhoods. Among White children (n = 556 725), 21.0% (n = 116 720) resided in very high opportunity neighborhoods and 14.9% (n = 82 941) in very low opportunity neighborhoods. Nearly half of Black children (45.5% [65 051 of 142 994]) and Hispanic children (44.6% [117 458 of 263 455]) lived in very low opportunity areas, while only 6.9% of Black children (9861 of 142 994) and 7.5% of Hispanic children (19 729 of 263 455) lived in very high opportunity neighborhoods. The proportion of urgent, emergency, and trauma cases increased as neighborhood opportunity decreased: among urgent cases (n = 148 431), 31.5% (n = 46 733) were from very low opportunity neighborhoods vs 12.6% (n = 18 712) from very high opportunity neighborhoods; among emergency cases (n = 326 908), 30.4% (n = 99 290) were from very low opportunity neighborhoods vs 15.8% (n = 51 497) from very high opportunity neighborhoods; and among trauma cases (n = 11 215), 32.5% (n = 3649) were from very low opportunity neighborhoods vs 10.4% (n = 1162) from very high opportunity neighborhoods.

**Table.** Characteristics of Study Participants by Neighborhood Opportunity Level^a^

Characteristic	Encounters, No./total No. (%) (N = 996 865)^b^				
	Very high	High	Moderate	Low	Very low
	159 457 (16.0%)	17 698 (17.9%)	179 024 (18.0%)	210 236 (21.1%)	269 450 (27.0%)
Age group					
Infant (<1 y)	29 433/209 736 (14.0)	37 269/209 736 (17.8)	39 459/209 736 (18.8)	46 217/209 736 (22.0)	57 358/209 736 (27.3)
Young child (≥1 to <5 y)	28 784/195 860 (14.7)	34 902/195 860 (17.8)	35 322/195 860 (18.0)	41 816/195 860 (21.3)	55 036/195 860 (28.1)
Child (≥5 to <13 y)	52 438/329 713 (15.9)	57 874/329 713 (17.6)	57 734/329 713 (17.5)	69 748/329 713 (21.2)	91 919/329 713 (27.9)
Adolescent (≥13 to <18 y)	48 802/261 556 (18.7)	48 653/261 556 (18.6)	46 509/261 556 (17.8)	52 455/261 556 (20.1)	65 137/261 556 (24.9)
Sex					
Female	71 870/441 584 (16.3)	79 999/441 584 (18.1)	79 611/441 584 (18.0)	92 451/441 584 (20.9)	117 653/441 584 (26.6)
Male	87 587/555 281 (15.8)	98 699/555 281 (17.8)	99 413/555 281 (17.9)	117 785/555 281 (21.2)	151 797/555 281 (27.3)
Race and ethnicity					
Hispanic	19 729/263 455 (7.5)	31 437/263 455 (11.9)	37 840/263 455 (14.4)	56 991/263 455 (21.6)	117 458/263 455 (44.6)
Non-Hispanic Asian	13 147/33 691 (39.0)	7074/33 691 (21.0)	5029/33 691 (14.9)	4441/33 691 (13.2)	4000/33 691 (11.9)
Non-Hispanic Black	9861/142 994 (6.9)	17 642/142 994 (12.3)	21 161/142 994 (14.8)	29 279/142 994 (20.5)	65 051/142 994 (45.5)
Non-Hispanic White	116 720/556 725 (21.0)	122 545/556 725 (22.0)	114 994/556 725 (20.7)	119 525/556 725 (21.5)	82 941/556 725 (14.9)
Length of stay, median (IQR), d	3 (2-6)	3 (2-6)	3 (2-6)	3 (2-6)	3 (2-7)
Admission type					
Elective	88 086/510 311 (17.3)	98 267/510 311 (19.3)	96 394/510 311 (18.9)	107 786/510 311 (21.1)	119 778/510 311 (23.5)
Urgent	18 712/148 431 (12.6)	25 667/148 431 (17.3)	26 232/148 431 (17.7)	31 087/148 431 (20.9)	46 733/148 431 (31.5)
Emergency	51 497/326 908 (15.8)	53 100/326 908 (16.2)	54 342/326 908 (16.6)	68 679/326 908 (21.0)	99 290/326 908 (30.4)
Trauma	1162/11 215 (10.4)	1664/11 215 (14.8)	2056/11 215 (18.3)	2684/11 215 (23.9)	3649/11 215 (32.5)
Insurance					
Commercial	119 333/436 540 (27.3)	105 106/436 540 (24.1)	82 726/436 540 (19.0)	73 543/436 540 (16.8)	55 832/436 540 (12.8)
Government: Medicaid, other	33 708/512 629 (6.6)	65 343/512 629 (12.7)	87 547/512 629 (17.1)	125 202/512 629 (24.4)	200 829/512 629 (39.2)
Self-pay	2112/17 141 (12.3)	3059/17 141 (17.8)	3091/17 141 (18.0)	4103/17 141 (23.9)	4776/17 141 (27.9)
Other payer	3749/26 013 (14.4)	4552/26 013 (17.5)	5030/26 013 (19.3)	6624/26 013 (25.5)	6058/26 013 (23.3)
Unknown	555/4542 (12.2)	638/4542 (14.0)	630/4542 (13.9)	764/4542 (16.8)	1955/4542 (43.0)
Preoperative complex chronic conditions^c^					
Cardiovascular	26 463/171 626 (15.4)	31 620/171 626 (18.4)	32 418/171 626 (18.9)	36 747/171 626 (21.4)	44 378/171 626 (25.9)
Gastrointestinal	21 761/143 124 (15.2)	26 312/143 124 (18.4)	27 328/143 124 (19.1)	30 870/143 124 (21.6)	36 853/143 124 (25.7)
Hematologic and immunologic	5810/39 470 (14.7)	6851/39 470 (17.4)	7072/39 470 (17.9)	8107/39 470 (20.5)	11 630/39 470 (29.5)
Neoplasms or malignant tumors	12 150/64 609 (18.8)	12 449/64 609 (19.3)	11 822/64 609 (18.3)	13 036/64 609 (20.2)	15 152/64 609 (23.5)
Metabolic	8265/52 034 (15.9)	9300/52 034 (17.9)	9431/52 034 (18.1)	11 009/52 034 (21.2)	14 029/52 034 (27.0)
Neurologic and neuromuscular	28 495/180 947 (15.7)	34 179/180 947 (18.9)	34 669/180 947 (19.2)	39 003/180 947 (21.6)	44 601/180 947 (24.6)
Renal and urologic	18 377/116 658 (15.8)	22 236/116 658 (19.1)	22 576/116 658 (19.4)	25 011/116 658 (21.4)	28 458/116 658 (24.4)
Respiratory	13 718/88 859 (15.4)	16 698/88 859 (18.8)	17 213/88 859 (19.4)	19 057/88 859 (21.4)	22 173/88 859 (25.0)
Procedural group					
Cardiovascular	17 230/108 413 (15.9)	19 940/108 413 (18.4)	20 368/108 413 (18.8)	23 049/108 413 (21.3)	27 826/108 413 (25.7)
Digestive and metabolic	33 251/223 548 (14.9)	37 463/223 548 (16.8)	38 429/223 548 (17.2)	46 794/223 548 (20.9)	67 611/223 548 (30.2)
Hematology and oncology	3491/19 985 (17.5)	3725/19 985 (18.6)	3562/19 985 (17.8)	4135/19 985 (20.7)	5072/19 985 (25.4)
Neonatology	5310/42 473 (12.5)	7572/42 473 (17.8)	8235/42 473 (19.4)	9560/42 473 (22.5)	11 796/42 473 (27.8)
Neurology	19 637/116 746 (16.8)	22 553/116 746 (19.3)	21 797/116 746 (18.7)	24 781/116 746 (21.2)	27 978/116 746 (24.0)
Orthopedics and joint disease	34 088/195 976 (17.4)	35 567/195 976 (18.1)	34 692/195 976 (17.7)	40 737/195 976 (20.8)	50 892/195 976 (26.0)
Other surgical	39 102/249 864 (15.6)	44 167/249 864 (17.7)	44 734/249 864 (17.9)	53 198/249 864 (21.3)	68 663/249 864 (27.5)
Respiratory	5403/27 042 (20.0)	5452/27 042 (20.2)	4902/27 042 (18.1)	5315/27 042 (19.7)	5970/27 042 (22.1)
Transplant	1945/12 818 (15.2)	2259/12 818 (17.6)	2305/12 818 (18.0)	2667/12 818 (20.8)	3642/12 818 (28.4)
Hospital volume					
High	116 812/716 227 (16.3)	129 869/716 227 (18.1)	126 796/716 227 (17.7)	149 275/716 227 (20.8)	193 475/716 227 (27.0)
Low	42 645/280 638 (15.2)	48 829/280 638 (17.4)	52 228/280 638 (18.6)	60 961/280 638 (21.7)	75 975/280 638 (27.1)
Year of surgery					
2012	10 646/81 777 (13.0)	13 528/81 777 (16.5)	12 969/81 777 (15.9)	16 812/81 777 (20.6)	27 822/81 777 (34.0)
2013	10 802/83 895 (12.9)	13 399/83 895 (16.0)	13 073/83 895 (15.6)	17 116/83 895 (20.4)	29 505/83 895 (35.2)
2014	10 824/82 262 (13.2)	13 051/82 262 (15.9)	13 248/82 262 (16.1)	16 977/82 262 (20.6)	28 162/82 262 (34.2)
2015	10 772/80 918 (13.3)	13 288/80 918 (16.4)	13 377/80 918 (16.5)	16 846/80 918 (20.8)	26 635/80 918 (32.9)
2016	11 153/78 838 (14.1)	13 564/78 838 (17.2)	13 429/78 838 (17.0)	17 096/78 838 (21.7)	23 596/78 838 (29.9)
2017	11 864/77 219 (15.4)	13 472/77 219 (17.4)	13 364/77 219 (17.3)	17 391/77 219 (22.5)	21 128/77 219 (27.4)
2018	12 555/77 174 (16.3)	13 943/77 174 (18.1)	14 064/77 174 (18.2)	16 916/77 174 (21.9)	19 696/77 174 (25.5)
2019	13 235/74 460 (17.8)	13 665/74 460 (18.4)	14 166/74 460 (19.0)	16 379/74 460 (22.0)	17 015/74 460 (22.9)
2020	12 462/69 071 (18.0)	13 352/69 071 (19.3)	13 504/69 071 (19.6)	14 484/69 071 (21.0)	15 269/69 071 (22.1)
2021	14 329/74 330 (19.3)	14 710/74 330 (19.8)	14 455/74 330 (19.4)	15 203/74 330 (20.5)	15 633/74 330 (21.0)
2022	13 829/72 955 (19.0)	14 468/72 955 (19.8)	14 406/72 955 (19.7)	14 982/72 955 (20.5)	15 270/72 955 (20.9)
2023	14 637/76 915 (19.0)	15 007/76 915 (19.5)	15 380/76 915 (20.0)	16 044/76 915 (20.9)	15 847/76 915 (20.6)
2024^d^	12 349/67 051 (18.4)	13 251/67 051 (19.8)	13 589/67 051 (20.3)	13 990/67 051 (20.9)	13 872/67 051 (20.7)
In-hospital mortality					
Died	1000/8893 (11.2)	1455/8893 (16.4)	1575/8893 (17.7)	1994/8893 (22.4)	2869/8893 (32.3)

^a^ Patient encounters for inpatient surgical procedures at a Children’s Hospital Association participating hospital from January 1, 2012, to September 30, 2024.

^b^ Percentages correspond to the row.

^c^ Binary variable indicating the presence of the condition.

^d^ Includes only first to third quartile encounters for the year.

A total of 71.8% of patients (n = 716 227) were treated at high-volume centers, and 28.2% (n = 280 638) at low-volume centers (Table). Patient distribution across neighborhood opportunity levels was similar between high- and low-volume hospitals. Among commercially insured children (n = 436 540), most lived in higher opportunity areas (119 333 [27.3%] very high, 105 106 [24.1%] high), while 55 832 (12.8%) lived in very low opportunity areas. In contrast, 39.2% of government-insured children (200 829 of 512 629) lived in very low opportunity areas and 6.6% (33 708 of 512 629) in very high opportunity areas. Preoperative complex chronic conditions were more common among children from lower opportunity neighborhoods. Among children who died postoperatively, 32.3% (2869 of 8893) were from very low opportunity neighborhoods and 11.2% (1000 of 8893) from very high opportunity neighborhoods.

### Postoperative Mortality and Neighborhood Opportunity

**Figure 1.**
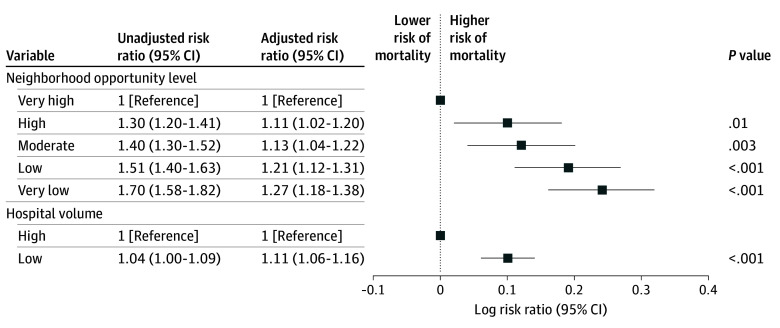
Unadjusted and Adjusted Risk Ratios of In-Hospital Postoperative Mortality Risk ratios for in-hospital postoperative mortality are shown for key exposures, including neighborhood opportunity and hospital volume. Unadjusted estimates represent crude associations, while adjusted models controlled for age, sex, race and ethnicity, admission type, length of stay, insurance type, procedural group, year of surgery, and preoperative complex chronic conditions. Error bars represent 95% CIs.

The overall in-hospital postoperative mortality rate was 0.9% (8893 children). Mortality was lowest among children living in very high opportunity neighborhoods (0.6% [1000 of 159 457]) and increased progressively as neighborhood opportunity decreased, reaching 1.1% (2869 of 269 450) among children from very low opportunity areas (eTable 1 in Supplement 1). Children living in high opportunity neighborhoods had an 11% higher risk of postoperative mortality compared with those living in very high opportunity neighborhoods (ARR, 1.11; 95% CI, 1.02-1.20; *P* = .01) (Figure 1). This risk increased progressively as neighborhood opportunity decreased, with a 13% higher risk among those from moderate opportunity neighborhoods (ARR, 1.13; 95% CI, 1.04-1.22; *P* = .003), a 21% higher risk among those from low opportunity neighborhoods (ARR, 1.21; 95% CI, 1.12-1.31; *P* < .001), and a 27% higher risk among children from very low opportunity neighborhoods (ARR, 1.27; 95% CI, 1.18-1.38; *P* < .001).

### Postoperative Mortality and Hospital Volume

Of the 49 hospitals included in this study, 24 were classified as high volume, having a total of 716 227 encounters, while 25 were low volume, having a total of 280 638 encounters. The mortality rate was higher for low-volume hospitals at 0.92% (n = 2583), compared with 0.88% (n = 6310) for high-volume hospitals (eTable 1 in Supplement 1). Low-volume hospitals had an 11% higher risk of postoperative mortality (ARR, 1.11; 95% CI, 1.06-1.16; *P* < .001) compared with high-volume hospitals (Figure 1).

### Adjusted Risk of Mortality by Neighborhood and Hospital Volume

**Figure 2.**
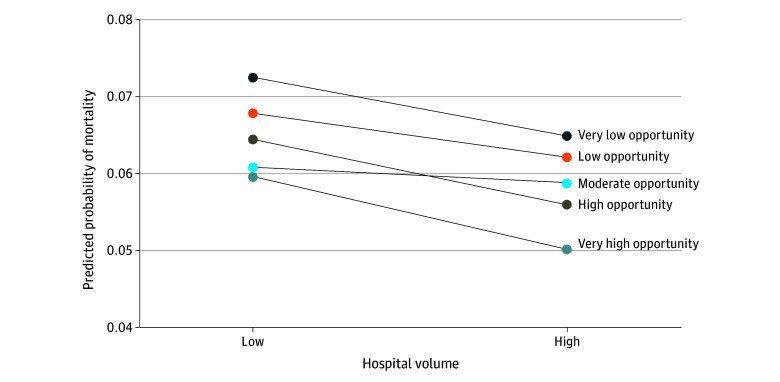
Estimated Mean In-Hospital Postoperative Mortality Rates Across Hospital Volume by Neighborhood Opportunity Level Predicted probabilities of in-hospital postoperative mortality were estimated using a generalized estimating equations model including an interaction term between hospital volume and neighborhood opportunity level. Mortality rates decreased with increasing hospital volume across all neighborhood opportunity levels. The decrease was more pronounced for children in very high opportunity neighborhoods and high opportunity neighborhoods, while reductions in mortality were smaller and more variable among children from lower opportunity neighborhoods.

**Figure 3.**
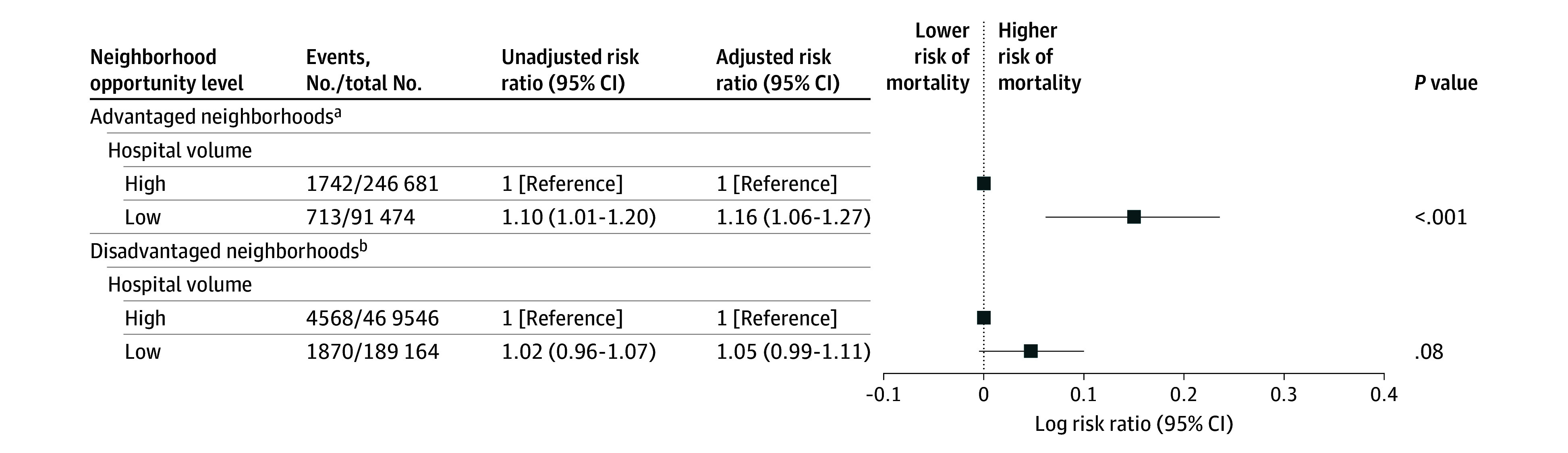
Association Between Hospital Volume and In-Hospital Postoperative Mortality, Stratified by Neighborhood Opportunity Level The adjusted model controlled for age, sex, race and ethnicity, admission type, length of stay, insurance type, procedural group, year of surgery, and preoperative complex chronic conditions. Error bars represent 95% CIs. ^a^Advantaged neighborhoods include neighborhoods with very high and high opportunities using Child Opportunity Index 3.0. ^b^Disadvantaged neighborhoods include neighborhoods with moderate, low, and very low opportunities using Child Opportunity Index 3.0.

Figure 1 shows that there was minimal change to the association between neighborhood opportunity and postoperative mortality in models that included hospital volume compared with models that did not include hospital volume (eTable 2 in Supplement 1). No significant interaction was observed between neighborhood opportunity and hospital volume on either the additive (RERI, −0.07; 95% CI, −0.19 to 0.04) or multiplicative scale (ARR, 0.93; 95% CI, 0.84-1.03; *P* = .14) (eTable 3 in Supplement 1**)**. The adjusted probability plot showed that mortality decreased with high volume across all opportunity levels, although the strength of this association varied (Figure 2). Among children from advantaged neighborhoods, treatment at low-volume hospitals was associated with a 16% higher risk of death compared with high-volume hospitals (ARR, 1.16; 95% CI, 1.06-1.27; *P* < .001) (Figure 3). However, among children from disadvantaged neighborhoods, the risk of mortality was no different for those treated at high-volume vs low-volume hospitals (ARR, 1.05; 95% CI, 0.99-1.11; *P* = .08).

### Temporal Trends in Postoperative Mortality

**Figure 4.**
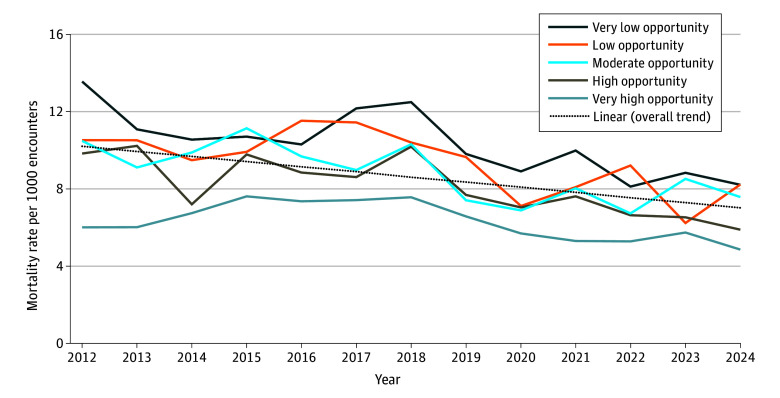
Temporal Trends in In-Hospital Postoperative Mortality Rates by Neighborhood Opportunity Level, 2012-2024 This graph illustrates mortality rates per 1000 surgical encounters from 2012 to 2024, categorized by neighborhood opportunity level. The dotted line represents the linear trend, which illustrates the overall pattern of mortality over the study period.

Overall, postoperative mortality decreased from approximately 10 deaths per 1000 encounters in 2012 to 6 per 1000 in 2024, representing a relative decrease of 35.2%; however, the temporal improvement varied by neighborhood opportunity levels. In very low opportunity neighborhoods, mortality rates declined from 13.6 to 8.2 per 1000 encounters, while in very high opportunity neighborhoods, it decreased from 6.0 to 4.9 per 1000 encounters (Figure 4). Postoperative mortality also declined in both high-volume and low-volume hospitals: from 9.5 per 1000 to 6.5 per 1000 in high-volume hospitals and from 12.2 per 1000 to 5.1 per 1000 in low-volume hospitals (eFigure 1 in Supplement 1).

### Sensitivity Analysis

Estimates from the model clustered by hospital ID were consistent with those clustered by patient ID, suggesting that findings were robust to potential within-hospital correlation (eFigure 2 in Supplement 1). In the primary analysis, E-values for exposure and hospital-volume ranged from 1.46 to 1.86, with lower 95% CI limits ranging from 1.16 to 1.64, suggesting that the observed associations are unlikely to be explained by unmeasured confounding (eFigure 3 in Supplement 1).

## Discussion

In this large retrospective analysis of a national administrative database, we found that area-level social drivers of health were associated with pediatric postoperative mortality. Specifically, children from neighborhoods with lower opportunity had a markedly higher risk of death after surgery compared with their peers from more advantaged neighborhoods. Although treatment at high-volume surgical centers was generally associated with lower mortality, our analysis revealed no evidence that hospital volume modified the association between lower opportunity neighborhoods and postoperative mortality risk. In other words, high-volume centers did not buffer the elevated risk faced by children from disadvantaged communities. These findings highlight the need to address upstream, community-level inequities as part of any meaningful strategy to improve surgical outcomes among children.

Although sociodemographic disadvantage is a known risk factor for premature death, our study is the first, to our knowledge, to directly associate neighborhood opportunity with postoperative mortality among children.^21^ When compared with children from very high opportunity neighborhoods, postoperative mortality was 11% to 27% greater for children from lower opportunity areas. Our data also showed that children from disadvantaged neighborhoods were disproportionately represented among urgent, emergency, and trauma cases, underscoring how delayed preventive care, barriers to timely treatment, and greater exposure to unsafe environments increase acuity at presentation.^22,23^ These patterns reflect broader neighborhood disadvantages, including chronic disease burden, environmental hazards, limited access to health care, fewer educational and economic opportunities, socioeconomic constraints, and food and housing insecurity.^24,25,26,27,28^ Addressing these disparities will require multifaceted strategies, such as embedding community health workers and nurse navigators to improve care coordination,^29^ expanding school-based and community-based preventive care programs, and partnering with local organizations to provide reliable transportation and nutrition support.^30^ Hospitals can also implement trauma-informed care pathways and strengthen referral systems to primary care for children at high risk. At the policy level, investment in neighborhood infrastructure and Medicaid coverage for perioperative care and wraparound services are essential steps toward narrowing the mortality gap.^31^

Central to the narrative of disparities in health care outcomes is the well-documented reality that patients who are socially disadvantaged are disproportionately cared for in lower-volume, lower-quality hospitals.^7,32,33,34,35,36^ Consistent with previous studies, we noted a strong inverse association between hospital volume and postoperative mortality risk.^34^ However, our findings show that hospital volume did not mitigate the elevated postoperative mortality risk for children from disadvantaged neighborhoods. This pattern mirrors that of major cardiovascular surgery, where higher hospital volume also does not offset the impact of social disadvantage.^37^ Taken together, these results highlight that hospital volume alone cannot overcome the mortality burden of social disadvantage—systemic change is needed to achieve equity in pediatric care.

Against this backdrop, the field has increasingly recognized the broader role of social drivers of health. Prior work demonstrates that addressing these factors can yield measurable improvements in patient outcomes and reduce health care costs.^38^ Our study demonstrates that children from low-opportunity neighborhoods remain at disproportionate risk, emphasizing the importance of centering this group in efforts to achieve equitable surgical access and outcomes. Concurrently with improvements in surgical quality and safety, we found that overall pediatric postoperative mortality has decreased approximately 35% in the past 12 years.^39,40,41^ The postoperative mortality gap between disadvantaged and very high opportunity neighborhoods has narrowed during this time, potentially reflecting increased attention to the social drivers of health.^42^ In addition, the development and validation of tools such as the Child Opportunity Index have increased awareness among health care professionals and researchers of the profound effect a child’s environment has on their health and well-being. Although these efforts represent meaningful progress toward equitable health outcomes, disparities in pediatric surgical mortality remain. Closing these gaps will require sustained, multisector investment in factors directly linked to surgical risk, including timely access to preventive and perioperative care, transportation to reduce cancellations and delays, nutrition programs to address malnutrition, safe housing to reduce trauma exposure, and educational supports to improve health literacy.

### Limitations

This study has some limitations. As with other database studies, our study is limited by lack of granularity. We do not have information about the severity of patients’ comorbidities, details of the surgeries, or insight on postoperative events that led to the child’s demise. Although we controlled for many potential confounders, unmeasured factors could still influence the results. However, the calculated E-value of 1.86 suggests that an unmeasured confounder would need to have a strong association with both the exposure and outcome to fully explain the observed results, suggesting that our findings are relatively robust to unaccounted confounding variables, and remain relevant. As with other large database studies using the Child Opportunity Index, the PHIS database limits address geocoding to the zip code level to preserve patient privacy.^8,43,44^ Although the zip code–level Child Opportunity Index has been validated and generally correlates with US Census tract–level measures, it may obscure within-area variation and misclassify neighborhood opportunity.^8^ This misclassification may differ by race and ethnicity, often overestimating opportunity for White children and underestimating it for Black and Hispanic children.^8^ Although race and ethnicity were included as covariates in adjusted models to reduce confounding, we acknowledge that this approach does not fully address potential bias from differential exposure misclassification. Our study used the PHIS database, which includes primarily freestanding children’s hospitals that are likely higher volume and better resourced than many smaller hospitals not represented in the PHIS database. This may limit the generalizability of our findings on hospital volume, as the differences observed within the PHIS database may underestimate disparities across the full spectrum of pediatric surgical care. Nevertheless, volume variation among PHIS hospitals was sufficient to demonstrate associations with postoperative mortality, suggesting that even within a network of predominantly high-volume centers, volume remains an important factor associated with outcomes. Finally, our characterization of a child’s neighborhood opportunity was static and did not account for factors such as residential mobility or shared households. However, research indicates that individuals generally stay within similar opportunity areas, even when families relocate or engage in coparenting.^45^

## Conclusions

Our cohort study underscores the complex association between area-level social drivers of health and pediatric postoperative mortality. Children from lower-opportunity neighborhoods faced significantly higher risks of in-hospital postoperative death, and this disparity persisted even at high-volume hospitals. These findings highlight that improving outcomes for socially disadvantaged children will require strategies beyond concentrating care in large centers. A comprehensive, equity-focused approach must include ensuring timely access to preventive and perioperative care, reducing transportation and scheduling barriers, addressing food and housing insecurity, and investing in neighborhood resources that support children’s health. Targeted action on these drivers is essential to narrowing the pediatric surgical mortality gap.

## Notes

Supplement 1. **eTable 1.** In-Hospital Postoperative Mortality (% of Total Encounters) by Neighborhood Opportunity Level, Hospital Volume, and Year **eTable 2.** Adjusted Risk Ratio for In-Hospital Postoperative Mortality (Not Adjusted for Hospital Volume) **eTable 3.** Interaction Between Hospital Volume and Neighborhood Opportunity on Risk of In-Hospital Postoperative Mortality **eFigure 1.** Temporal Trends in In-Hospital Mortality Rates per 1000 Encounters (2012-2024), by Hospital Volume **eFigure 2.** Sensitivity Analysis of Adjusted Risk Ratio for In-Hospital Postoperative Mortality (Clustered by Hospital ID) **eFigure 3.** Sensitivity Analysis Showing E-Values for the Association Between In-Hospital Postoperative Mortality and Exposures in the Primary Analysis

Supplement 2.
Data Sharing Statement
