# Supplementary material for: Neighborhood Opportunity, Hospital Volume, and Pediatric Postoperative Mortality
Source: JAMA Netw Open. 2025 Nov 12;8(11):e2543017. doi: 10.1001/jamanetworkopen.2025.43017 (PMC12612942; doi:10.1001/jamanetworkopen.2025.43017)
Supplement: Supplement 1. — eTable 1. In-Hospital Postoperative Mortality (% of Total Encounters) by Neighborhood Opportunity Level, Hospital Volume, and Year eTable 2. Adjusted Risk Ratio for In-Hospital Postoperative Mortality (Not Adjusted for Hospital Volume) eTable 3. Interaction Between Hospital Volume and Neighborhood Opportunity on Risk of In-Hospital Postoperative Mortality eFigure 1. Temporal Trends in In-Hospital Mortality Rates per 1000 Encounters (2012-2024), by Hospital Volume eFigure 2. Sensitivity Analysis of Adjusted Risk Ratio for In-Hospital Postoperative Mortality (Clustered by Hospital ID) eFigure 3. Sensitivity Analysis Showing E-Values for the Association Between In-Hospital Postoperative Mortality and Exposures in the Primary Analysis [file jamanetwopen-e2543017-s001.pdf]

Supplementary Online Content

Tay S, Mpody C, Tobias JD, Willer BL. Neighborhood opportunity, hospital volume, and pediatric postoperative mortality. *JAMA Netw Open.* 2025;8(11):e2543017. doi:10.1001/jamanetworkopen.2025.43017

eTable 1. In-Hospital Postoperative Mortality (% of Total Encounters) by Neighborhood Opportunity Level, Hospital Volume, and Year

eTable 2. Adjusted Risk Ratio for In-Hospital Postoperative Mortality (Not Adjusted for Hospital Volume)

eTable 3. Interaction Between Hospital Volume and Neighborhood Opportunity on Risk of In-Hospital Postoperative Mortality

eFigure 1. Temporal Trends in In-Hospital Mortality Rates per 1000 Encounters (2012-2024), by Hospital Volume

eFigure 2. Sensitivity Analysis of Adjusted Risk Ratio for In-Hospital Postoperative Mortality (Clustered by Hospital ID)

eFigure 3. Sensitivity Analysis Showing E-Values for the Association Between In-Hospital Postoperative Mortality and Exposures in the Primary Analysis

This supplementary material has been provided by the authors to give readers additional information about their work.

eTable 1. In-Hospital Postoperative Mortality (% of Total Encounters) by Neighborhood Opportunity Level, Hospital Volume, and Year

	**No. of deaths/ No. of encounters**	**Mortality rate** (%)
**Neighborhood opportunity level**		
Very High	1000/159 457	0.63
High	1455/178 698	0.81
Moderate	1575/179 024	0.88
Low	1994/210 236	0.95
Very Low	2869/269 450	1.06
**Year**		
2012	887/81 777	1.08
2013	828/83 895	0.99
2014	756/82 262	0.92
2015	813/80 918	1.00
2016	772/78 838	0.98
2017	780/76 439	1.01
2018	804/77 174	1.04
2019	622/74 460	0.84
2020	497/69 071	0.72
2021	583/74 330	0.78
2022	528/72 955	0.72
2023	553/76 915	0.72
2024	470/67 051	0.70
**Hospital volume**		
High-volume (No. of hospitals, n= 24)	6310/716 227	0.88
Low-volume (No. of hospitals, n= 25)	2583/280 638	0.92

© 2025 Tay S et al. *JAMA Network Open.*

eTable 2. Adjusted Risk Ratio for In-Hospital Postoperative Mortality (Not Adjusted for Hospital Volume)

	Adjusted	
	**Risk ratio (95% CI)**	**P-value**
**Neighborhood opportunity level**		
Very High	1 [Reference]	
High	1.12 (1.02-1.20)	.01
Moderate	1.14 (1.04-1.22)	.002
Low	1.21 (1.12-1.31)	<.001
Very Low	1.27 (1.18-1.38)	<.001

The model controlled for age, sex, race and ethnicity, admission type, length of stay, insurance type, procedural group, year of surgery, and preoperative complex chronic conditions.

© 2025 Tay S et al. *JAMA Network Open.*

eTable 3. Interaction Between Hospital Volume and Neighborhood Opportunity on Risk of In-Hospital Postoperative Mortality

	Advantaged Neighborhoods (Very High or High COI)		Disadvantaged Neighborhoods (Very Low, Low, or Moderate COI)	
	**aRR (95% CI)**	**P-value**	**aRR (95% CI)**	**P-value**
**Hospital volume**				
High-volume	1 [Reference]		1.16 (1.09-1.23)	<.001
Low-volume	1.17 (1.07-1.28)	<.001	1.26 (1.15-1.36)	<.001

Measure of interaction on additive scale: RERI (95% CI) = -0.07 (-0.19-0.04).
Measure of interaction on multiplicative scale: RR (95% CI) = 0.93 (0.84-1.03); P = .14
Abbreviations: aRR, adjusted risk ratio; CI, Confidence Interval; RERI, relative excess risk due to interaction.
The adjusted model accounts for age, sex, race and ethnicity, admission type, length of stay, insurance type, preoperative complex chronic conditions, procedural group, and year of surgery.

© 2025 Tay S et al. *JAMA Network Open.*

eFigure 1. Temporal Trends in In-Hospital Mortality Rates per 1000 Encounters (2012-2024), by Hospital Volume

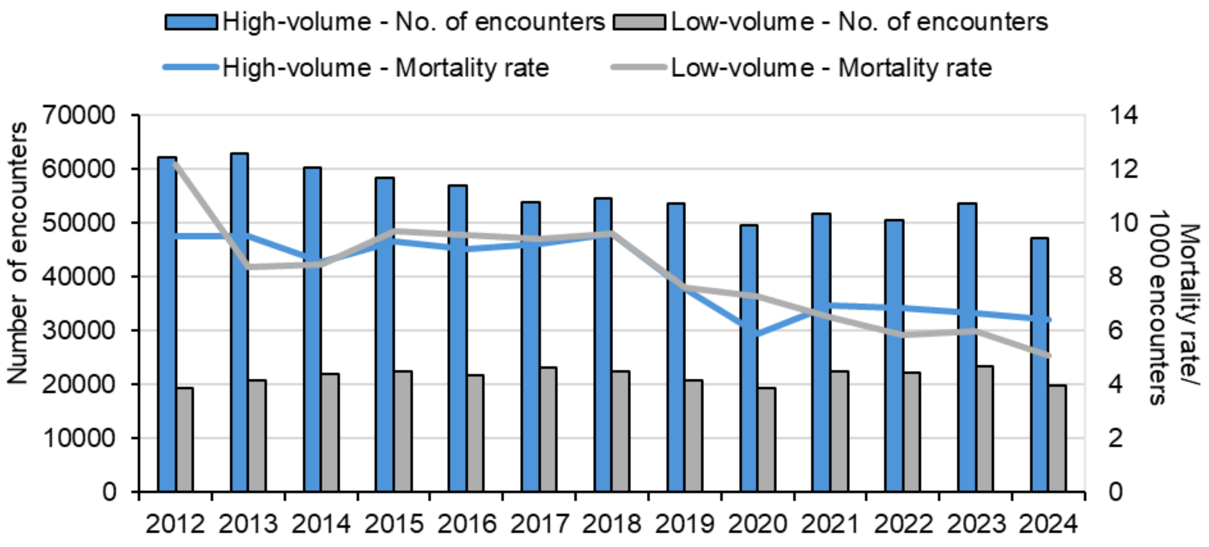

© 2025 Tay S et al. *JAMA Network Open.*

eFigure 2. Sensitivity Analysis of Adjusted Risk Ratio for In-Hospital Postoperative Mortality (Clustered by Hospital ID)

	Adjusted Risk ratio (95% CI)	P-value
**Neighborhood opportunity level**		
Very High	1 [Reference]	
High	1.10 (1.02-1.20)	.01
Moderate	1.12 (1.04-1.22)	.004
Low	1.20 (1.12-1.30)	<.001
Very Low	1.27 (1.17-1.37)	<.001
**Hospital volume**		
High-volume	1 [Reference]	
Low-volume	1.10 (1.05-1.15)	<.001

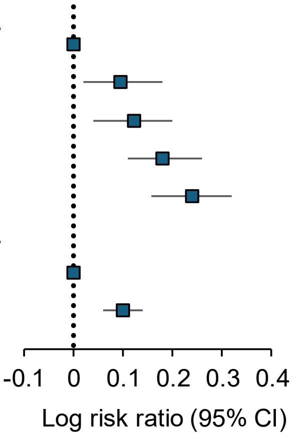

The model controlled for age, sex, race and ethnicity, admission type, length of stay, insurance type, procedural group, year of surgery, and preoperative complex chronic conditions. Error bars represent 95% confidence intervals.

© 2025 Tay S et al. *JAMA Network Open.*

eFigure 3. Sensitivity Analysis Showing E-Values for the Association Between In-Hospital Postoperative Mortality and Exposures in the Primary Analysis

	Adjusted risk ratio (95% CI)	E-value for point estimate	E-value for confidence interval
**Neighborhood opportunity level**			
Very High	1 [Reference]		
High	1.11 (1.02-1.20)	1.46	1.16
Moderate	1.13 (1.04-1.22)	1.51	1.24
Low	1.21 (1.12-1.31)	1.71	1.49
Very Low	1.27 (1.18-1.38)	1.86	1.64
**Hospital volume**			
High-volume	1 [Reference]		
Low-volume	1.11 (1.06-1.16)	1.46	1.31

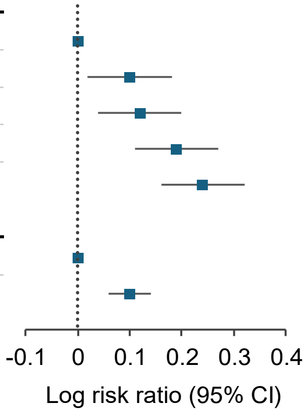

The model controlled for age, sex, race and ethnicity, admission type, length of stay, insurance type, procedural group, year of surgery, and preoperative complex chronic conditions.

© 2025 Tay S et al. *JAMA Network Open.*
