# Supplementary material for: Neighborhood Opportunity, Hospital Volume, and Pediatric Postoperative Mortality
Source: JAMA Netw Open. 2025 Nov 12;8(11):e2543017. doi: 10.1001/jamanetworkopen.2025.43017 (PMC12612942; doi:10.1001/jamanetworkopen.2025.43017)
Supplement: Supplement 2. — Data Sharing Statement [file jamanetwopen-e2543017-s002.pdf]

Data Sharing Statement

Tay. Neighborhood Opportunity, Hospital Volume, and Pediatric Postoperative Mortality. *JAMA Netw Open*. Published November 12, 2025. doi:10.1001/jamanetworkopen.2025.43017

Data
Data available: No

Additional Information
Explanation for why data not available: The data used in this study are from the Pediatric Health Information System (PHIS), which is subject to data use agreements that prohibit public sharing of individual-level data. Researchers may apply for access directly through the Children's Hospital Association.
